# Taking the Book from the Bookshelf: Masked Constituent Priming Effects from Compound Words and Nonwords

**DOI:** 10.5334/joc.11

**Published:** 2018-01-26

**Authors:** Elisabeth Beyersmann, Yvette Kezilas, Max Coltheart, Anne Castles, Johannes C. Ziegler, Marcus Taft, Jonathan Grainger

**Affiliations:** 1 Department of Cognitive Science and ARC Centre of Excellence in Cognition and its Disorders, Macquarie University, AU; 2 Aix-Marseille Univ and CNRS, Laboratoire de Psychologie Cognitive, FR; 3 School of Psychology, University of New South Wales, AU

**Keywords:** compound word processing, embedded words, masked priming, lexical decision

## Abstract

Recent evidence from visual word recognition points to the important role of embedded words, suggesting that embedded words are activated independently of whether they are accompanied by an affix or a non-affix. The goal of the present research was to more closely examine the mechanisms involved in embedded word activation, particularly with respect to the “edge-alignedness” of the embedded word. We conducted two experiments that used masked priming in combination with lexical decision. In Experiment 1, monomorphemic target words were either preceded by a compound word prime (e.g., *textbook-BOOK/textbook-TEXT*), a compound-nonword prime (e.g., *pilebook-BOOK/textpile-TEXT*), a non-compound nonword prime (e.g., *pimebook-BOOK/textpime-TEXT*) or an unrelated prime (e.g., *textjail-BOOK/jailbook-TEXT*). The results revealed significant priming effects, not only in the compound word and compound-nonword conditions, but also in the non-compound nonword condition, suggesting that embedded words (e.g., *book*) were activated independently of whether they occurred in combination with a real morpheme (e.g., *pilebook*) or a non-morphemic constituent (e.g., *pimebook*). Priming in the compound word condition was greater than in the two nonword conditions, indicating that participants benefited from the whole-word representation of real compound words. Constituent priming occurred independently of whether the target word was the first or the second embedded constituent of the prime (e.g., *textbook-BOOK* vs. *textbook-TEXT*). In Experiment 2, significant priming effects were found for edge-aligned embedded constituents (e.g., *pimebook-BOOK*), but not for mid-embedded (e.g., *pibookme-BOOK*) or the outer-embedded constituents (e.g., *bopimeok-BOOK*), suggesting that edge-alignedness is a key factor determining the activation of embedded words.

For many years, researchers have examined the mechanisms involved in reading morphologically complex words. It is widely agreed upon that complex words are automatically decomposed into morphemic subunits during the initial stages of visual word recognition (as initially suggested by Taft & Forster, 1975). It has been proposed that the segmentation of morphologically complex words is based on a mechanism that identifies and strips off the affix, which then in turn allows the identification of the stem morpheme. However, this hypothesis has recently been challenged by Grainger and Beyersmann (2017) suggesting that embedded words (rather than affixes) represent the primary reading units that initiate morphological segmentation. The focus of our present study was therefore on the role of the stem, rather than the affix, during early visual word recognition. In particular, our goal was to shed more light on the mechanisms involved in activating words embedded in compound words and compound nonwords.

Evidence obtained with various paradigms suggests that the processing of the constituent lexemes in compound words, such as *text* and *book* in *textbook*, contributes to the overall process of compound word recognition (for a review, see Marelli & Luzzatti, 2012). The masked priming paradigm (Forster & Davis, 1984) has played a key role in revealing the mechanisms involved in recognizing morphologically complex words. By presenting complex prime stimuli very briefly, such that the only visible stimuli are morphologically simple targets (e.g., *farmer-FARM*), participants are generally unaware of the nature of the priming manipulation, deeming the strategic use of prime-target relations highly unlikely (e.g., Rastle, Davis, Marslen-Wilson, & Tyler, 2000; Rastle, Davis, & New, 2004). Many studies have investigated the processing of affixed words (for reviews, see Amenta & Crepaldi, 2012; Rastle & Davis, 2008), and the results suggest that these are rapidly segmented into stems and affixes at pre-lexical stages of word recognition, independently of semantics. However, very few masked priming studies have investigated the processing of compound words. Compound words differ from derived words in one obvious but fundamental aspect: stems are combined with stems, rather than stems with affixes. Any structural compound priming effects can therefore not be attributed to the traditional affix-stripping hypothesis. Furthermore, practically all of these studies have investigated priming of compound word targets with constituent primes (e.g., *farm-farmhouse*; Crepaldi, Rastle, Davis, & Lupker, 2013; Duñabeitia, Marín, Avilés, Perea, & Carreiras, 2009; Shoolman & Andrews, 2003), which is the direct opposite of the conditions tested in studies examining non-compound derivations, namely, complex words priming simplex targets (e.g., farmer-farm; Beyersmann, Ziegler, et al., 2016; Rastle & Davis, 2008; Rastle et al., 2000; Rastle et al., 2004). Given the visibility of the compound word target, these studies cannot answer questions concerning the automaticity of morphological processing during visual word recognition.

To our knowledge, only one published study has examined masked priming by compound primes on constituent targets. Fiorentino and Fund-Reznicek (2009) found equivalent priming from transparent (*teacup*) and opaque (*honeymoon*) compound words on both the first and second constituent (*teacup-TEA/teacup-CUP*). No priming was found in the non-compound control condition (*window-WIN*). These results suggest that compound words are automatically segmented into their morphemic constituents without any influence from semantics, and are therefore consistent with much prior masked priming research on affixed words. Importantly however, the mechanism that has been described to account for pseudo-affixed word priming effects (e.g., *corner-corn*; Rastle et al., 2004), suggesting that affixes are rapidly chunked/stripped-off, can obviously not account for the type of structural compound priming effects observed by Fiorentino and Fund-Reznicek (2009). Instead, these findings indicate that the activation of embedded stems is only successful if the written word can be exhaustively decomposed into its morphemic consituents (as is the case *tea-cup* and *honey-moon*) and so such activation would not occur with non-compound words such as *window*. But what precisely is the mechanism that prevents the activation of the embedded word *win* in *window*?

One theory that provides an answer to this important question is the edge-aligned embedded word activation theory proposed by Grainger and Beyersmann (2017), by which embedded words are activated at both ‘edges’ of the letter string (i.e., when the string begins or ends with the embedded word). The activation of embedded words is based on a purely non-morphological process of mapping input letters onto existing whole-word representations in the orthographic lexicon. Grainger and Beyersmann’s account predicts that the reading system rapidly identifies embedded words independently of morphological structure (e.g., *tea* and *cup* in *teacup, honey* and *moon* in *honeymoon*, and *win* in *window*). Due to lateral inhibition between the whole word (e.g., *teacup, honeymoon, window*, etc.) and the embedded words (e.g., *tea, cup honey, moon, win*, etc.), the activation of the embedded words is initially hindered. However, due to the *principle of full decomposition*, the identification of embedded words is facilitated when the whole letter string can be completely divided into constituent morphemes. For example, *teacup* can be divided into *tea* and *cup, honeymoon* can be divided into *honey* and *moon*, whereas for *window* the principle of full decomposition fails. Grainger and Beyersmann (2017) propose that the principle of full decomposition provides a boost in activation to the embedded word that helps overcome the lateral inhibition between the embedded word and the whole letter string, which explains why significant priming is observed in the *teacup* and *honeymoon* conditions, but not in the *window* condition.

In the present study, we build on Fiorentino and Fund-Reznicek’s initial work in a further exploration of the mechanisms involved in reading compound words. If it is true that the morphological segmentation of compound words is uniquely based on structural information, one would expect to observe priming not only from real compound word primes (*textbook*), but also compound-nonword primes (*pilebook*). Since compound-nonwords cannot be mapped onto an existing representation in the lexicon, any evidence for priming in these conditions would therefore provide clear-cut evidence for pre-lexical activation of embedded words. Moreover, if it is true that ‘edge-alignedness’ is a key factor determining the activation of embedded words (Grainger & Beyersmann, 2017), we would expect significant and equivalent priming for words when embedded in initial and final string position (Experiment 1), but not when embedded in mid-string position (Experiment 2).

An additional goal of our study was to compare priming from compound-nonwords (*pilebook*) and non-compound nonwords (*pimebook*). The embedded word activation hypothesis of Grainger and Beyersmann (2017) predicts that the activation of the embedded word is not hindered by the lexical representation of the nonword prime in these conditions. That is, one would expect to see priming not only in the compound nonword condition (*pilebook- BOOK*), but also in the non-compound nonword condition (*pimebook- BOOK*). Since *pimebook* is not lexically represented, it does not compete with the lexical representation of the embedded word *book* and is therefore able to facilitate priming in this condition. Indeed, several recent masked priming studies have reported comparable magnitudes of priming to words embedded in affixed and non-affixed nonword primes (Beyersmann, Casalis, Ziegler, & Grainger, 2015; Beyersmann, Cavalli, Casalis, & Colé, 2016; Beyersmann & Grainger, 2017; Hasenäcker, Beyersmann, & Schroeder, 2016; Morris, Porter, Grainger, & Holcomb, 2011). Beyersmann, Casalis, et al. (2015), for instance, reported significant priming effects not only when the target was preceded by a suffixed word prime (*banker-BANK*) or a suffixed nonword prime (*bankify-BANK*), but also when it was preceded by a non-suffixed nonword prime (*bankord-BANK*). Moreover, when participants were split into two groups depending on their individual vocabulary and spelling proficiencies, it was found that high proficiency participants showed robust priming in all three prime conditions, whereas low proficiency participants showed significantly reduced non-suffixed priming compared to the two suffixed conditions (see also Andrews & Lo, 2013, for comparable findings). Given this influence of language proficiency on morphological priming effects, we also measured the language proficiency of the participants in the present study.

Importantly, embedded word priming effects have not always been supported by the literature. For instance, an influential study by (Longtin & Meunier, 2005) reported greater priming from suffixed nonword primes (*bankify-BANK*) than non-suffixed nonword primes (*bankord-BANK*), thus providing evidence in favor of a morpho-orthographic segmentation mechanism that is insensitive to semantics but sensitive to surface morphological structure (similar results, although less clear cut, are also reported by McCormick, Rastle, & Davis, 2009). These findings conflict with the above reported embedded word priming studies, and thus further highlight the need for a close examination of the underlying embedded word activation mechanisms.

## Experiment 1

### Method

#### Participants

Eighty students from Macquarie University, all English native speakers, participated for course credit. Prior to testing, all participants received information about the study and signed a written consent.

#### Materials

We selected a list of 52 compound words (*textbook*) from the CELEX lexical database (Baayen, Piepenbrock, & van Rijn, 1993), using a number of selection criteria. All compound-constituents were either nouns, verbs or adjectives (we excluded *anybody*) and always mono-morphemic (we excluded *glassmaking*, as well as pseudo-affixed constituents such as the *butter* of *buttermilk* and the *body* of *bodyguard*). Compound words were always transparent (e.g., we excluded partially opaque compounds like *keyboard*, where the most frequent usage of *key* is *door-key*). The compound’s second constituent never appeared as a first constituent (since we used *textbook*, we excluded *bookshelf*).

Based on the compound words, we created two stimulus sets (Appendix A). In Set 1, compound primes were paired with a target that was the compound’s second constituent (*textbook-BOOK*). In Set 2, compound primes were paired with a target that was the compound’s first constituent (*textbook-TEXT*). Compound word primes were nearly identical in Sets 1 and 2, except that 5 compound words had to be replaced in Set 2 to avoid target repetition. Target words of Sets 1 and 2 were matched on word frequency, length and orthographic neighborhood (descriptive statistics are reported in Table 1).

**Table 1:** Descriptive statistics for primes and targets used in Experiment 1.

word initial embeddings			
	2nd constituent of compound word	2nd constituent of compound nonword	target word

word frequency	196.82	137.13	145.69
number of syllables	1.10	1.06	1.10
number of phonemes	3.37	3.33	3.27
number of letters	4.27	4.25	4.13
orthographic neighbourhood	8.73	8.02	7.40
phonological neighbourhood	19.44	17.04	16.50
**word final embeddings**			
	**1st constituent of compound word**	**1st constituent of compound nonword**	**target word**

word frequency	139.19	118.95	172.31
number of syllables	1.10	1.04	1.08
number of phonemes	3.21	3.29	3.33
number of letters	4.12	4.12	4.21
orthographic neighbourhood	7.06	7.88	8.92
phonological neighbourhood	16.79	15.71	19.38

In addition to the compound words, we selected 52 compound-nonwords, 52 non-compound nonwords, and 52 unrelated nonwords. In Set 1, compound-nonwords were created by replacing the first constituent of the compound word with a novel word constituent (*pilebook-BOOK*), non-compounds by replacing 1–2 letters within the first constituent of the compound word (*pimebook-BOOK*), and unrelated nonwords were created by replacing the second constituent of the compound prime with an orthographically unrelated mono-morphemic constituent (*textjail-BOOK*). In Set 2, primes were created by replacing the second constituents, using the same principles as in Set 1 (*textpile-TEXT/textpime-TEXT/jailbook-TEXT*). Constituents of compound words and compound-nonwords were of the exact same length (*textbook* vs. *pilebook*). Constituents of compound-nonword primes were selected such that they never occurred in first or second constituent position in real compound primes. Constituents of compound words and compound nonwords were matched on frequency, number of letters, number of phonemes, number of syllables, orthographic neighborhood, and phonological neighbourhood (see Table 1). All item specific variables were retrieved from the LEXIQUE database (New, Pallier, Brysbaert, & Ferrand, 2004).

For the purpose of the lexical decision task, we included 52 nonword targets (*NESH, NUNE, etc*.), which were orthographically legal and pronounceable and matched on length to the real-word targets. Each nonword target was preceded by four different primes, which were structured in the same way as the primes preceding the word targets. In Set 1, primes were created by replacing the second constituents of the compound word, compound-nonwords, and non-compound conditions with the nonword target (*textnesh-NESH/pilenesh-NESH/pimenesh-NESH*), compared to an unrelated control (*textbrav-NESH*). In Set 2, primes were created by replacing the first constituents (*nunebook-NUNE/nunepile-NUNE/nunepime-NUNE/wostbook-NUNE*). To avoid target repetition, we created four counterbalanced lists.

#### Procedure

Stimuli were presented in the centre of an LED computer screen using DMDX software (Forster & Forster, 2003). Each trial consisted of a 500-ms forward mask of hash keys, then a 50-ms prime in lowercase, then the uppercase target. The target remained present until the response was made or until 3 seconds had elapsed. Participants were instructed to respond as quickly and accurately as possible.

#### Measures of individual differences

In addition to the masked primed lexical decision task, each participant was assessed with a vocabulary, spelling proficiency, reading efficiency, and a reading proficiency test.

***Vocabulary.*** The Shipley-2 (Shipley, Gruber, Martin, & Klein, 2009) was used as a vocabulary test, consisting of 40 items which increased in difficulty as the test progressed. For each item, a target word was presented in uppercase (*TALK*), and participants were asked to circle one word out of four alternatives that had a corresponding meaning (*draw, eat, speak, sleep*). Participants were given up to 10 minutes to complete the 40 items.

***Spelling proficiency.*** The spelling test comprised 50 words (8–10 letters long) selected from Burt and Tate (2002). First, words were verbally presented. Then they were included in a carrier sentence to clarify their meaning, and then repeated. Participants were asked to correctly spell each word.

***Reading efficiency.*** Reading efficiency was assessed using the sight word efficiency subtest and the phonemic decoding subtest of the Test of Word Reading Efficiency (TOWRE; Torgesen, Wagner, & Rashotte, 1999), Form A. Both subtests measured the number of words/nonwords participants could name in 45 seconds (for each test), with increasing difficulty as the test progressed. The sight word subtest was administered first, followed by the phonemic decoding subtest. The sight word list included 104 words, and the phonemic subtest 63 nonwords. Participants were asked to read as many words/nonwords as quickly as they could. The score for each subtest was the number of words/nonwords read correctly within 45 seconds.

Both subtests were scored following the protocol outlined by Torgeson et al. (1999). Because the TOWRE is an American normed test, we made some modifications to the scoring guidelines for the phonemic decoding subtest. Specifically, in addition to the Torgeson et al. (1999) protocol, we scored participants’ nonword responses in the phonemic subtest as correct if it was deemed correct following the Australian child comparison data reported by Marinus, Kohnen, and McArthur (2013).

***Reading proficiency.*** An extended version of the Castles and Coltheart Test 2 (Castles et al., 2009) was administered to assess the participants’ ability to sound out words and their whole word recognition ability. The extended test included an additional 45 items that were selected to be challenging for adult readers. Participants read aloud 55 regular words (*cat, oust*), 55 irregular words (*yacht, heir*), and 55 nonwords (*gop, spogsoub*), which were presented one at a time, in a mixed order. The degree of difficulty gradually increased as the task went on.

### Results and Discussion

Lexical decisions to word targets were analysed as follows. Incorrect responses were removed from the reaction time (RT) analysis (2.1% of all data). Inverse RTs (1/RT) were calculated for each participant to correct for RT distribution skew and used throughout the analyses. There were no reaction times smaller than 300 ms or larger than 3000 ms that had to be excluded from the analyses. RTs and error rates are presented in Table 2.

**Table 2:** Table 2 shows mean lexical decision times (ms) and error rates (%) for word targets in Experiment 1, averaged across participants. Standard errors are shown in parentheses.

Condition	Reaction times	Error Rates	Example

*First Constituent*			
compound word	544 (10)	1.3 (0.5)	*textbook-TEXT*
compound-nonword	558 (11)	2.5 (0.8)	*textpile-TEXT*
non-compound nonword	557 (11)	2.5 (0.9)	*textpime-TEXT*
unrelated	569 (10)	2.7 (0.8)	*jailbook-TEXT*
*Second Constituent*			
compound word	545 (10)	1.5 (0.5)	*textbook-BOOK*
compound-nonword	557 (12)	1.2 (0.4)	*pilebook-BOOK*
non-compound nonword	548 (9)	3.1 (0.9)	*pimebook-BOOK*
unrelated	569 (14)	1.9 (0.5)	*textjail-BOOK*

We used linear mixed-effects modelling to perform the main analyses (Baayen, 2008; Baayen, Davidson, & Bates, 2008). Fixed and random effects were only included if they significantly improved the model’s fit in a backward stepwise model selection procedure. Models were selected using chi-squared log-likelihood ratio tests with regular maximum likelihood parameter estimation. Trial order was included to control for longitudinal task effects such as fatigue or habituation. To assess whether the obtained effects were modulated by individual differences in vocabulary, spelling or reading proficiency, the vocabulary, spelling and reading proficiency scores were standardized. A composite measure of reading (ReadZ) was calculated by averaging the standardized scores of the two reading tests. The composite ReadZ measures and standardized spelling (SpellZ) and vocabulary scores (VocabZ) were highly correlated (ReadZ vs. SpellZ: *r* = .684; ReadZ vs. VocabZ: *r* = .480; SpellZ vs. VocabZ: *r* = .454). Thus, the first measure of individual differences used was one that captured the common variance between ReadZ, SpellZ and VocabZ (LanguageProficiencyZ), and which had high positive correlations with all three measures (reading: *r* = .908; spelling: *r* = .842; vocabulary: *r* = .737). Three additional measures of individual differences were used: one that captured the difference between ReadZ and SpellZ (ReadSpellDiffZ), one that captured the difference between ReadZ and VocabZ (ReadVocabDiffZ), and one that captured the difference between SpellZ and VocabZ (SpellVocabDiffZ). ReadSpellDiffZ showed opposite directions of relationship with reading (*r* = .114) and spelling (*r* = –.647). ReadVocabDiffZ showed opposite directions of relationship with reading (*r* = .312) and vocabulary (*r* = –.684). SpellVocabDiffZ showed opposite directions of relationship with spelling (*r* = .522) and vocabulary (*r* = –.522).

**Figure 1:**
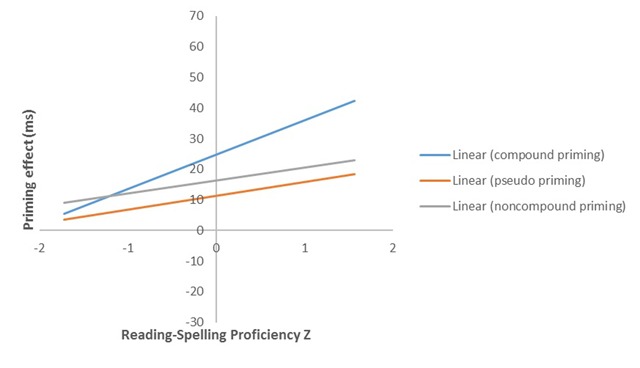
Priming effects for targets preceded by compound word, compound-nonword, and non-compound nonword primes (relative to the unrelated control condition), as a function of individual differences in reading and spelling proficiency. Positive proficiency scores represent individuals who are better readers than spellers. Negative proficiency scores represent individuals who are better spellers than readers.

A linear mixed-effects model, as implemented in the lme4 package (Bates, Maechler, Bolker, & Walker, 2014) in the statistical software R (Version 3.0.3; R Development Core Team, 2008), was created with seven fixed effects factors (primetype: compound word, compound-nonword, non-compound nonword, unrelated; LanguageProficiencyZ; ReadSpellDiffZ; ReadVocabDiffZ; SpellVocabDiffZ; embedded word position: first constituent, second constituent; trial order), their interactions, and two random effects factors (random intercepts for subjects and items). All continuous variables were centred (i.e. trial order, LanguageProficiencyZ, ReadSpellDiffZ, ReadVocabDiffZ, and SpellVocabDiffZ). Both here and throughout, values with regard to *p* were determined using the package lmerTest (Kuznetsova, Brockhoff, & Christensen, 2014). The model was refitted after excluding data-points whose standardized residuals were larger than 2.5 in absolute value (2.6%; see Baayen, 2008). RT analyses revealed a significant priming effect in the compound word condition (*t* = 6.63, *p* < .001), in the compound-nonword condition (*t* = 3.48, *p* < .001) and in the non-compound nonword condition (*t* = 4.22, *p* <. 001), relative to the unrelated control. Moreover, priming was significantly greater in the compound word than in the compound-nonword or non-compound nonword conditions (*t* = 2.39, *p* = .017; *t* = 6.63, *p* <. 001), but there was no significant difference between priming in the compound-nonword and non-compound nonword conditions (*t* = 0.75, *p* = .451). LanguageProficiencyZ, SpellVocabDiffZ, and ReadVocabDiffZ did not yield any significant effects or interactions. However, RT analyses revealed that priming in the compound word condition was significantly modulated by SpellReadDiffZ (Figure 1), indicating that participants whose reading skills were more proficient than their spelling skills showed more priming in the compound word condition relative to the unrelated condition (*t* = 2.30, *p* = .021). There was also a significant effect of trial order (*t* = 2.47, *p* = .014). No other effects were significant.

Error analyses followed the same logic as the RT analyses. We applied a binomial variance assumption to the trial-level binary data using the function *glmer* as part of the R-package *lme4*. There was a significant effect of trial order (*z* = 2.83, *p* = .005) and a significant effect of SpellVocabDiffZ (*z* = 2.11, *p* = .035), suggesting that participants with higher spelling than vocabulary skills produced fewer errors. No other effects were significant.

The results of Experiment 1 are clear-cut. Priming is obtained independently of whether the embedded target is accompanied by a real morpheme (e.g., *textbook-BOOK* and *pilebook-BOOK*) or by a non-morphemic ending (e.g., *pimebook-BOOK*), and independently of position (i.e. in initial or final string position: *textbook-BOOK/textbook-TEXT*). The priming effect seen in the *pimebook-BOOK* condition is consistent with previous embedded word priming studies (Beyersmann et al., 2015; Beyersmann, Cavalli, et al., 2016; Beyersmann & Grainger, 2017; Hasenäcker et al., 2016; Morris et al., 2011). Moreover, greater priming was obtained in the compound word condition relative to the two nonword prime conditions, which was particularly pronounced in individuals who were better readers than spellers (we return to this point in the General Discussion). Most importantly, the results of Experiment 1 suggest that the activation of embedded words is based on an entirely non-morphological process, which is consistent with Grainger and Beyersmann’s (2017) embedded word activation hypothesis. Embedded words are activated simply by mapping the letters of the input string onto existing whole-word representations in the orthographic lexicon, independently of morphological structure.

However, if it is true that the activation of embedded words is based on a clearly non-morphological process, one crucial question to ask is why Fiorentino and Fund-Reznicek (2009) failed to observe priming in the *window-WIN* condition? We hypothesise that the absence of priming in this condition can be explained by lateral inhibition between the lexical representations of *window* and *win*. Of course, there would also be lateral inhibition between opaque compound words (e.g., *butterfly*) and their embedded words (e.g., *butter*). Here, however, the presence of the second constituent (e.g., *fly*) would boost the activation of the first constituent (*butter*) and therefore produce priming (Grainger & Beyersmann, 2017).

The goal of Experiment 2 was to further manipulate the position of the embedded words. In particular, our goal was to more closely examine the principle of ‘edge-alignedness’ of embedded word processing. Grainger and Beyersmann (2017) proposed that embedded words receive the greatest bottom-up support when they are edge-aligned, because stems occur less often internally and because edge-aligned words benefit from having a space next to one letter which can be used as an anchor point during orthographic processing (Fischer-Baum, Charny, & McCloskey, 2011). Experiment 2 was designed to test this hypothesis.

## Experiment 2

The aim of Experiment 2 was to compare edge-aligned embedded word priming (e.g., *pimebook-BOOK*), to a mid-embedded condition (e.g., *pibookme-BOOK*) and an outer-embedded condition (e.g., *bopimeok-BOOK*). If the activation of embedded words is simply due to lower-level orthographic letter-to-word mappings, we would expect that the amount of priming should be determined by the predicted amount of orthographic overlap between prime and target. The predicted amount of orthographic overlap differs depending on different orthographic coding schemes (cf. Match Calculator software, Version 1.9, programmed by Colin J. Davis).^1^ The binary open-bigram model (Grainger & van Heuven, 2003) generates comparable proportions of orthographic overlap for the edge-aligned and mid conditions, and relatively less overlap in the outer condition. This model thus predicts significantly less priming in the outer condition compared to the edge-aligned and mid conditions. The overlap open-bigram model (Grainger, Granier, Farioli, Van Assche, & van Heuven, 2006), the SERIOL model (Whitney & Cornelissen, 2008), and the SOLAR model (Davis, 2010) generate comparable proportions of orthographic overlap in all three conditions, and therefore predict comparable amounts of priming across all three conditions. However, if it is true that the reading system gives priority to letters that are contiguous with either the first or the last letter of the string (Grainger & Beyersmann, 2017), we would expect to see significantly more priming in the edge-aligned condition compared to the mid and outer control conditions, with comparable magnitudes of priming between the mid and outer conditions.

### Method

#### Participants

Forty-seven students from the University of New South Wales, all English native speakers, participated for course credit.

#### Materials

We used the exact same non-compound nonwords with word final edge-aligned embeddings (e.g., *pimebook-BOOK*) as in Experiment 1 (see Appendix A). In addition, we created a mid-embedded condition (e.g., *pibookme-BOOK*) and an outer-embedded condition (e.g., *bopimeok-BOOK*). Some primes were slightly changed to avoid having letters of the target being repeated in the second constituent of the prime. Finally, we used the items of the unrelated control condition of Experiment 1, but the first constituent was replaced with the non-morphemic first constituent of the non-compound prime (e.g., *fraltdraft* instead of *motordraft*). All items are listed in Appendix B.

#### Procedure

The procedure of the masked priming task was identical to the one used in Experiment 1. Since Experiment 1 showed no evidence for the influence of vocabulary, reading and morphological awareness on masked non-compound priming, we did not assess individual proficiency measures in Experiment 2.

### Results and Discussion

Lexical decisions to word targets were analysed as follows. Incorrect responses were removed from the reaction time (RT) analysis (5.4% of all data). Inverse RTs (1/RT) were calculated for each participant to correct for RT distribution skew and used throughout the analyses. Reaction times smaller than 300 ms or larger than 3000 ms were excluded from the analyses (1.2% of the data). The data from two participants and one item were excluded, because error rates were above 20%. RTs and error rates are presented in Table 3.

**Table 3:** Table 3 shows mean lexical decision times (ms) and error rates (%) for word targets in Experiment 2, averaged across participants. Standard errors are shown in parentheses.

Condition	Reaction times	Error Rates	Example

edge-aligned	552 (12)	2.8 (0.9)	*pimebook-BOOK*
outer	561 (11)	6.8 (1.1)	*bopimeok-BOOK*
mid	563 (12)	5.2 (1.0)	*pibookme-BOOK*
unrelated	564 (12)	5.6 (0.9)	*pimejail-BOOK*

As in Experiment 1, we used linear mixed-effects modelling to perform the main analyses (Baayen, 2008; Baayen et al., 2008). A linear mixed-effects model was created with two fixed effects factors (primetype: edge-aligned, mid, outer, unrelated; trial order) and two random effects factors (random intercepts for subjects and items). All continuous variables were centred. The model was refitted after excluding data-points with standardized residuals larger than 2.5 in absolute value (2.5%; see Baayen, 2008). RT analyses revealed a significant priming effect in the edge-aligned embedded word condition (*t* = 2.44, *p* = .015), but not in the mid-embedded condition (*t* = 0.24, *p* = .811) or the outer embedded condition (*t* = 0.38, *p* = .708), relative to the unrelated control. Moreover, the results revealed that priming was significantly greater in the edge-aligned condition than in the mid or outer conditions (*t* = 2.69, *p* = .007; *t* = 2.05, *p* = .040), but there was no significant difference between priming in the mid and outer conditions (*t* = 0.61, *p* = .539). There was also a significant effect of trial order (*t* = 2.96, *p* = .003). No other effects were significant.

Error analyses revealed a similar pattern as the RT analyses. There was a significant priming effect in the edge-aligned embedded word condition (*z* = 2.05, *p* = .041), but not in the mid-embedded condition (*z* = 0.14, *p* = .888) or the outer embedded condition (*t* = 1.24, *p* = .216), relative to the unrelated control. Moreover, the results revealed that priming was significantly greater in the edge-aligned condition than in the mid or outer conditions (*z* = 2.17, *p* = .030; *z* = 3.17, *p* = .002), but there was no significant difference between priming in the mid and outer conditions (*z* = 1.10, *p* = .271). No other effects were significant.

The results of Experiment 2 confirm the second constituent priming effects found with non-compound nonword primes (e.g., *pimebook-BOOK*) in Experiment 1. Crucially, the significant priming effects found in this condition in Experiment 2 contrast with the non-significant priming effects seen with primes that share the same number of letters with targets but are either edge-aligned but non-contiguous (e.g., *bopimeok-BOOK*) or contiguous but non-edge-aligned (e.g., *pibookme-BOOK*). The first result is evidence that orthographic overlap in itself is not sufficient to generate priming effects, and the second result is evidence that having the target word embedded in the prime is not sufficient to generate priming effects. The complete set of results points to edge-alignment and lexical status of the letters shared by prime and target as two key factors driving the priming effects seen in Experiment 1.

## General Discussion

The primary goal of our study was to investigate embedded word activation processes operating during the processing of compound words and compound nonwords by using masked priming combined with the lexical decision task. In Experiment 1, significant priming effects relative to unrelated primes were observed for compound word primes (*textbook-BOOK/textbook-TEXT*), compound nonword primes (*pilebook-BOOK/textpile-TEXT*), and non-compound nonword primes (*pimebook-BOOK/textpime-TEXT*), and these priming effects did not differ for the first and second constituents. In Experiment 2, significant priming effects were found in the edge-aligned embedded word condition (e.g., *pimebook-BOOK*), but not in the mid-embedded condition (e.g., *pibookme-BOOK*) or the outer-embedded condition (e.g., *bopimeok-BOOK*).

The first key finding of Experiment 1 is the equivalent priming seen for compound nonword primes (*pilebook-BOOK*) and non-compound nonword primes (*pimebook-BOOK*). This replicates the embedded word priming effects previously obtained with derived nonword primes (e.g., *flexify* vs. *flexint*; Beyersmann et al., 2015; Morris et al., 2011) and thus points to the importance of embedded word activation mechanisms that operate independently of whether the embedded word occurs in combination with a real word (*book* in *pilebook*) or a nonword (*book* in *pimebook*). Contrary to Beyersmann et al. (2015), however, we failed to observe any influence of language proficiency on the non-compound nonword priming effects. Importantly, Experiment 2 demonstrates that priming is only obtained for edge-aligned embedded words (e.g., *book* in *pimebook*) and not for mid-embedded (e.g., *pibookme*) or outer-embedded positions (e.g., *bopimeok*), suggesting that the reading system prioritizes activation of embedded words in edge-aligned position. A straightforward explanation for the important role of edge-alignedness during printed word processing is that spaces surrounding written words may provide privileged anchor points for letter-word mappings (for a more detailed outline of this proposal, see Grainger & Beyersmann, 2017; see also Fischer-Baum et al., 2001, for a related proposal of the “both-edges” coding scheme).

The second key finding is that the magnitude of priming was the same whether the target word was the first or the second constituent of the prime (*textbook-BOOK* vs. *textbook-TEXT*). This result is compatible with previous studies investigating compound word processing (Crepaldi et al., 2013; Duñabeitia, Laka, Perea, & Carreiras, 2009; Duñabeitia, Marín, et al., 2009; Libben, Gibson, Yoon, & Sandra, 2003; Monsell, 1985; Sandra, 1990; Shoolman & Andrews, 2003; Zwitserlood, 1994), and suggests that both constituents of a compound word contribute equally to lexical access (but see Taft & Forster, 1976; Taft, Xu, & Li, 2017, who suggest that the first constituent is more important). Unlike prefixes and suffixes, embedded words are not subject to positional constraints, and this can explain why the identification of stem morphemes is position independent (Crepaldi et al., 2013). This finding constitutes important evidence against any sequential beginning-to-end processing bias that might influence complex word recognition (for converging evidence, see also Bowers, Davis, & Hanley, 2005). Our results also converge with previous evidence for embedded word activation mechanisms in monomorphemic nonwords (e.g., wish in dwish; Davis & Taft, 2005; Taft et al., 2017). In line with our present findings, Taft et al. (2017) suggest that edge-alignedness is an important factor underlying embedded word processing (i.e., initial and final consonants have priority in assigning letters to their position), whereas priming is not expected to arise for a mid- or outer-embedded words because of the disruption to the onset + vowel + coda structure of the target.

Experiment 1 revealed a third key result, namely that greater priming was obtained in the compound word condition relative to the two nonword prime conditions. This suggests that the whole-word representation of the compound word prime contributed to priming effects. The fact that Fiorentino and Fund-Reznicek (2009) found no difference between transparent and opaque compound word primes suggests that the difference between compound word and compound-nonword primes seen in our study is driven by whole-word form representations of the compound word and not by morpho-semantic representations. However, our observation that priming in the compound word condition was particularly pronounced in those participants who were better readers than spellers (Figure 1), suggests that future research should examine the role of language proficiency in determining the relative size of transparent and opaque compound word priming before ruling out a role for morpho-semantic representations. Highly relevant with respect to this possibility are the results reported by Andrews and Lo (2013), who found that individuals with higher proficiency in spelling relative to vocabulary showed stronger priming from opaque derived word primes (*corner-CORN*). In line with Andrews and Lo (2013), our present findings suggest that it is participants’ relative *spelling* proficiency that modulates priming. In other words, those individuals who were relatively poor spellers tend to rely more heavily on whole-word processing, which was also the case in Andrews and Lo (2013).

Our results concerning the effects of language proficiency are in line with the hypothesis that participants who are better readers than spellers place more emphasis on direct access to whole-word orthographic representations from print compared with morpho-orthographic segmentation processes. These participants would therefore benefit more from the lexical representation of real compound words (*textbook*), presumably because they would be particularly proficient in rapidly mapping input letter strings onto their corresponding whole-word representations. On the other hand, those participants who were better spellers than readers would be less efficient in mapping a complete letter string onto its whole-word representation and therefore rely to a greater extent on morpho-orthographic segmentation mechanisms.

Importantly, the present data provide further support for embedded word activation mechanisms operating at the level of whole-word representations. More precisely, our results allow us to rule-out an explanation of embedded word priming effects in terms of lower-level letter overlap between the prime and the target. If orthographic overlap, independently of whether the overlapping letters form a word or not, were the factor driving embedded word priming effects, then we should have observed similar priming from the non-contiguous primes (e.g., *bopimeok-BOOK*) in Experiment 2. Of course, it could be argued that having non-contiguous orthographic overlap is not an appropriate comparison, and that a more appropriate comparison might involve contiguous letters in so-called partial primes (e.g., *pimehant-ELEPHANT*).^2^ Although we agree that future experimentation should investigate this possibility, we would also point out that failure to find a partial priming effect in these conditions would not provide conclusive evidence that embedded word priming is lexically driven, since the partial prime shares four letters out of eight with the target, whereas the embedded word prime shares four letters out of four. On the other hand, finding significant priming with such partial primes would force us to re-consider our word-based interpretation of the present constituent priming effects. Finally, it has been shown that greater masked priming effects are observed for embedded words with many morphological family members compared to embedded words with no morphological family members (Beyersmann & Grainger, 2017), which is also suggestive of a lexical locus of the embedded word priming effect.

Taken together, the present findings, in addition to evidence obtained from prior research using different paradigms (e.g., Andrews, Miller, & Rayner, 2004; Hyönä, Bertram, & Pollatsek, 2004; Pollatsek, Hyönä, & Bertram, 2000; Zwitserlood, 1994), are consistent with parallel dual-route models that allow simultaneous access to both whole-word representations of compound words and the representations of their embedded morphemes (Baayen & Schreuder, 1999; Bertram & Hyönä, 2003; Diependaele, Sandra, & Grainger, 2009). However, it is also possible that the additional facilitation in the real compound condition in Experiment 1 comes from morphologically-mediated activation of the whole-word representation, rather than from a parallel whole-word processing route. Most importantly, our data suggest that embedded words (*book*) are activated when embedded at the edges of the letter string, independently of whether they occur in combination with a real morpheme (*pilebook*) or a non-morphemic constituent (*pimebook*). Our findings therefore point to the importance of embedded word activation mechanisms during complex word processing, in line with Grainger and Beyersmann’s (2017) recent proposal.

## Data Accessibility Statement

Could JoC please help me to make the data available on their website? If this is possible, I will provide the data in excel format via email, so that JoC can make these available online.

## Additional File

The Additional file for this article can be found as follows:

Data Files 1 and 2. Primes and targets used in Experiments 1 and 2. DOI: https://doi.org/10.6084/m9.figshare.5797338

## Footnotes

This application is available at Colin Davis’ website: http://www.pc.rhul.ac.uk/staff/c.davis/utilities/matchcalc/index.htm.

We thank an anonymous reviewer for this suggestion.
